# Advancing posaconazole quantification analysis with a new reverse-phase HPLC method in its bulk and marketed dosage form

**DOI:** 10.12688/f1000research.132841.2

**Published:** 2023-06-27

**Authors:** Annamalai Rama, Induja Govindan, Srinivas Hebbar, Abhishek Chaturvedi, Usha Rani, Anup Naha

**Affiliations:** 1 Department of Pharmaceutics, Manipal College of Pharmaceutical Sciences, Manipal Academy of Higher Education, Manipal, Karnataka, 576104, India; 2 Division of Biochemistry, Department of Basic Medical Sciences, Manipal Academy of Higher Education, Manipal, Karnataka, 576104, India; 3 Department of Social Health and Innovation, Prasanna School of Public Health, Manipal Academy of Higher Education, Manipal, Karnataka, 576104, India

**Keywords:** Posaconazole, HPLC, Analytical method, Validation, Quality control

## Abstract

**Introduction**: Posaconazole is a widely used antifungal drug, and its accurate quantification is essential for quality control and assessment of its pharmaceutical products. This study aimed to develop and validate a reverse-phase high-performance liquid chromatography (HPLC) analytical method for quantifying Posaconazole in bulk and dosage form.

**Methods**: The HPLC method was developed and validated based on International Conference on Harmonisation (ICH) guidelines. The developed method was then applied to quantify Posaconazole in a marketed tablet formulation. The method's specificity, linearity, precision, accuracy, robustness, and stability were evaluated.

**Results**: The developed HPLC method showed good linearity over a 2-20 μg/mL concentration range. The percentage recovery of Posaconazole from the bulk and marketed formulations was found to be 99.01% and 99.05%, respectively. The intra-day and inter-day precisions were less than 1%, and the method was stable under different conditions. The HPLC method was successfully applied to quantify Posaconazole in the marketed formulation.

**Conclusion**: The developed and validated HPLC method is reliable and efficient for analyzing Posaconazole in bulk and dosage forms. The method's accuracy, precision, specificity, linearity, robustness, and stability demonstrate its effectiveness. The method can be used for the quality control and assessment of Posaconazole-containing pharmaceutical products.

## Introduction

Posaconazole is an antifungal medication that inhibits the synthesis of ergosterol, a vital component of the fungal cell membrane, ultimately causing cell death. It is effective against a wide range of fungal pathogens, making it a popular treatment for various fungal infections. Accurate and precise quantification of posaconazole in dosage forms is crucial for ensuring proper dosing and therapeutic efficacy, quality control, and pharmacokinetic studies. However, existing methods for measuring posaconazole can be expensive, time-consuming, or unreliable. ^1^^–^^10^

High-performance liquid chromatography (HPLC) is a commonly used analytical technique for quantifying drugs in dosage forms. This study aims to develop and validate a new HPLC analytical method that is quick, sensitive, robust, cheap, and reliable for estimating posaconazole in bulk and dosage form. ^11^^–^^23^

The HPLC method involves,

▪ Preparing a solution of the active ingredient from the dosage form,
▪ Injecting it into the HPLC system, and
▪ Separating the different components based on their interactions with the stationary phase.

Posaconazole is detected using a UV-Vis detector, which measures the absorbance of posaconazole in the eluent. The peak area or height is then correlated to the concentration of posaconazole in the sample using a standard calibration curve. ^24^^–^^34^

The newly developed method was optimized by adjusting chromatographic conditions, including the mobile phase composition, column type, and detection wavelength. Validation was performed in accordance with International Conference on Harmonization (ICH) Q2 R1 guidelines, ensuring the method's accuracy, precision, and specificity. The method was successfully used to estimate posaconazole in its bulk and dosage forms. ^35^^–^^49^

Following CONSORT guidelines for reporting randomized trials increased the transparency of our study and improved the quality of our reporting, contributing to the overall usefulness of our findings. Our new analytical method has significant implications for drug delivery, including more accurate and efficient monitoring of posaconazole levels in patients, increasing overall drug efficacy and safety.

Overall, this manuscript presents the development and validation of a new HPLC analytical method for estimating posaconazole in bulk and dosage form, which will provide valuable insights into analytical method development and validation for drug delivery and contribute to the ongoing efforts to improve patient outcomes. ^50^^–^^63^

## Methods

A HPLC Grade Milli-Q water (Milli Q Direct-Q 3 UV Water Purification System, Merck, United States), Analytical Weighing Balance (BSA224S-CW, Sartorius, Germany), UV Spectrophotometer (UV 1800, Shimadzu, Japan) was used in the study. Posaconazole was received as ex-gratis from Lupin Healthcare limited. Ortho-phosphoric acid (88%) was from Merck Ltd. (Mumbai, India). HPLC grade Acetonitrile (MeCN) and methanol (purity, min 99.8%), was procured from Merck Ltd. (Mumbai, India). A Nylon membrane filter of 0.45 μm was obtained from HiMedia Pvt. Ltd (Mumbai, India). Phenomenex Hyperclone C18 column (5 μm particle size, 100 Å, 250 mm × 4.6 mm id) was procured from Phenomenex (Hyderabad, India), Other reagents and solvents utilized for the method development and validation were of analytical or HPLC grade.

The Shimadzu LC-2010CHT high-performance liquid chromatography model was used in the current research investigation. The device was equipped with a dual-wavelength ultraviolet detector, a column oven, and an autosampler from Shimadzu. The data acquisition of the chromatograms produced was done using LC Solution software version 5.57. A Hyperclone C18 column (250 mm x 4.6 mm in diameter, particle size 5 microns) from Phenomenex, USA. The mobile phase was filtered through a Millipore glass filter using 0.22-micron pore-size nylon membrane filter paper and connected to a glass vacuum filtration unit. The filtered mobile phase was then sonicated in a GT Sonic Professional Ultrasonic Cleaner)GT-2013QTS, GT Sonic, China) for 10 minutes to remove any air bubbles. The pH of the buffer was determined using a pH meter and a glass electrode (μ pH System 361, Systronics, India).

### Selection of wavelength

Posaconazole was weighed and mixed with spectroscopy grade methanol to prepare the stock solution (10 mg in 10 mL). A series of concentrations (5 μg/mL, 10 μg/mL, 15 μg/mL, 20 μg/mL, 30 μg/mL) were prepared from the stock solution, and a UV spectrophotometer determined their absorbance maxima. The absorbance maxima were then used for the HPLC method development. ^13^^,^^64^^–^^73^

### Selection of Stationary and Mobile Phase

Choosing the right stationary and mobile phase is critical in HPLC method development. The underlying protocol for the selection of Stationary Phase and Mobile Phase is reposited in the Protocols.io repository which can be accessed by following these references. ^74^^,^^75^ To develop the method, the Phenomenex Hyperclone C18 column was selected as the stationary phase, and Acetonitrile and Methanol (as Organic Phase) and 10mM Phosphate buffer (pH 6.8) (Aqueous Phase) were chosen as the mobile phase. The stock solution was prepared in Acetonitrile, and an isocratic mode of elution was employed for the method. The injection volume was set to 20 microliters, and the detection wavelength was selected based on the absorbance maxima determined by UV. The stationary phase was maintained at 25°C, while the flow rate of the mobile phase varied between 0.8 and 1.2 mL per minute. From the stock solution, 1 μg/mL was prepared and used in the analysis.

10 mg of Posaconazole was weighed accurately and transferred to a 10 ml volumetric flask. The volume was made up with Acetonitrile to prepare the stock solution. From the stock solution 1 μg/mL was prepared. Isocratic mode of elution was opted for the method. Injection volume was set to 20 microliters and the detection wavelength was selected based on the results of the determination of absorbance maxima by UV. Stationary phase was maintained at 25 ^o^C. Flow rate of the Mobile Phase was varied between 0.8 mL to 1.2 mL per minute.

After the initial method development trials were conducted, an optimized trial was carried forward for analytical method validation according to ICH guidelines. ^76^^–^^94^

### Validation of the optimized HPLC method

The validation of an analytical method is critical for ensuring that the method is appropriate and reliable for its intended usage. In this study, the optimized analytical technique was subjected to a series of validation experiments recommended by the International Conference on Harmonization (ICH) Q2(R1) guidelines in order to evaluate its performance under various situations. ^53^^,^^88^^,^^89^^,^^95^^–^^125^

### Specificity

An analytical method's capability to accurately measure the analyte despite the presence of diluents or excipients in the sample is referred to as “specificity.” To assess the specificity of the optimized analytical method, three replicates of a blank solution (diluent), Posaconazole (1 μg/mL), and a commercial formulation (equivalent to 1 μg/mL of Posaconazole) were introduced into the HPLC system. The resulting chromatograms were then examined for any interference from the diluents or excipients at the retention time of the Posaconazole.

### Linearity

Ensuring linearity is critical to analytical method development, as it guarantees accurate analyte quantification across a broad range of concentrations. To assess linearity, a range of serial concentrations, spanning from 0.1 to 32 μg/mL, were prepared from a standard Posaconazole drug solution using the mobile phase ratio as a diluent. Quintuplicate injections were made for each concentration, and the resulting peak areas (in mV-min) were plotted against their respective concentrations (in μg/mL) to create a linear regression graph. From this graph, the intercept and slope were calculated via a linear regression equation.

### Accuracy

The accuracy of an analytical method is determined by its ability to produce results that are close to the true or accepted value of the measured concentration. To evaluate the accuracy of the developed method, three concentrations from the linearity range (2 μg/mL, 4 μg/mL, and 8 μg/mL) were analyzed in sextuplicate. Intra-day accuracy was assessed by analyzing sextuplicate injections of all three concentrations twice on the same day (at 09:00 and 21:00 hours), while inter-day accuracy was evaluated by analyzing duplicate injections of all three concentrations over three consecutive days. The mean percentage recovery was calculated for each concentration to determine the method's accuracy.

### Sensitivity

Sensitivity is a crucial characteristic of an analytical method, as it determines the ability to detect small amounts of the analyte. In this study, the method's sensitivity was evaluated by determining Posaconazole's detection limit (DL) and quantitation limit (QL). The DL is defined as the lowest concentration of Posaconazole that can be detected with reliability and accuracy, while QL is the lowest concentration that can be measured with accuracy and precision. A lower DL and QL indicate a more sensitive method with the ability to detect and quantify low concentrations of the analyte.

The DL and QL are calculated based on the residual standard deviation of the regression line and its slope.

The formula is

$$\mathrm{DL}=3.3*\frac{\sigma }{S}$$

$$\mathrm{QL}=10*\frac{\sigma }{S}$$

Where, σ = residual standard deviation of the regression line

S = slope of the regression line

### Precision

Precision is a critical parameter in evaluating the reliability and reproducibility of an analytical method. It assesses the agreement between multiple measurements of the same homogeneous sample under the same experimental conditions. In this study, the precision of the developed analytical method was determined by analyzing four quality control concentrations of Posaconazole, including the quantitation limit, the lower quality control concentration (three times the quantitation limit), higher quality control concentration (70% of the highest concentration from the linearity concentration), and middle-quality control concentration (the mean of the lower and higher quality control concentrations). Sextuplicate injections of all quality control solutions were analyzed twice on the same day at two different times (9:00 and 21:00) to assess intra-day precision. Additionally, duplicate injections of all quality control concentrations were analyzed for inter-day precision for three consecutive days. The peak area for each concentration and the percentage relative standard deviation (%RSD) were calculated to evaluate the method's inter- and intra-day precision.

### Robustness

Robustness is a critical factor in assessing the stability and reliability of an analytical method. It ensures that minor variations in operating conditions do not significantly affect the method's performance. In this study, the robustness of the developed analytical method was evaluated by injecting a Posaconazole concentration of 2 μg/mL three times while varying certain conditions such as the acetonitrile ratio in the mobile phase (%), column oven temperature (°C), wavelength (nm), flow rate (mL/min), injection volume (μL), and pH of the aqueous phase. The responses were monitored for any changes, and each condition's peak area and retention time were analyzed to calculate the % RSD.

### System suitability

System suitability is an essential aspect of the analytical method validation that assesses the performance of the entire analytical system to ensure that it is suitable for the intended analysis. This includes the evaluation of various parameters, such as peak area and retention time (Rt). To determine system suitability for the developed analytical method, a Posaconazole concentration of 1μg/mL was injected in sextuplicate, and the peak area and Rt for each injection were analyzed. The %RSD of these parameters was calculated to assess the overall performance of the analytical system. This helps ensure that the analytical system performs consistently and reliably, which is crucial for obtaining accurate and precise analytical results.

### Application of the developed analytical method for quantification of posaconazole in the marketed product

The developed HPLC method is considered a valuable tool only after its practical application to quantify Posaconazole in dosage forms. Posaconazole was analyzed in the marketed tablet formulation (Picasa GR 100 mg Tablet, Manufactured by: MSN Laboratories, India, and Marketed by: Intas Pharmaceuticals Limited, India, Expiry Month and Year is April 2024) to test the method's practical feasibility. The marketed formulation's standard stock solution was prepared by accurately weighing 12.6 mg (equivalent to 10 mg of Posaconazole), transferring it to a 10 mL volumetric flask, and filling the volume with the mobile phase. Further dilutions were done with the mobile phase to obtain the desired concentration (1 μg/mL). The validated method was used to analyze the desired concentration, and the Rt and peak area were measured. The amount of Posaconazole in the desired concentration was calculated by comparing the peak areas. ^33^^,^^126^^–^^134^

## Results

### Selection of wavelength

**Figure 1.**
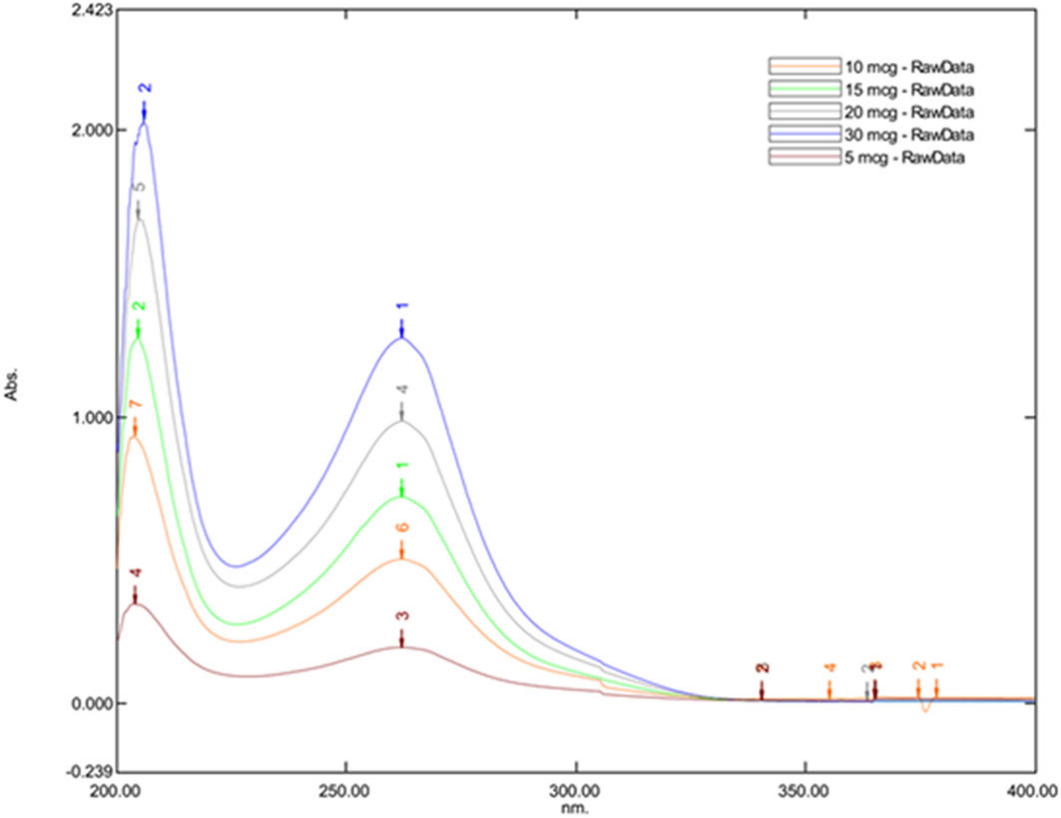
UV spectrum of Posaconazole to determine its absorption maxima in different concentrations.

The maximum absorbance was observed at 262.20 nm for all Posaconazole concentrations, leading to its selection as the optimal wavelength for subsequent HPLC analysis. This was evident with the Figure 1 which represents the UV spectrum of Posaconazole to determine its Absorption Maxima in different concentrations.

### Initial trials

Thirteen different chromatograms were obtained by altering the ratios of the organic and mobile phases, as well as the flow rate, during the experimental trials. These chromatograms are depicted below.

**Figure 2.**
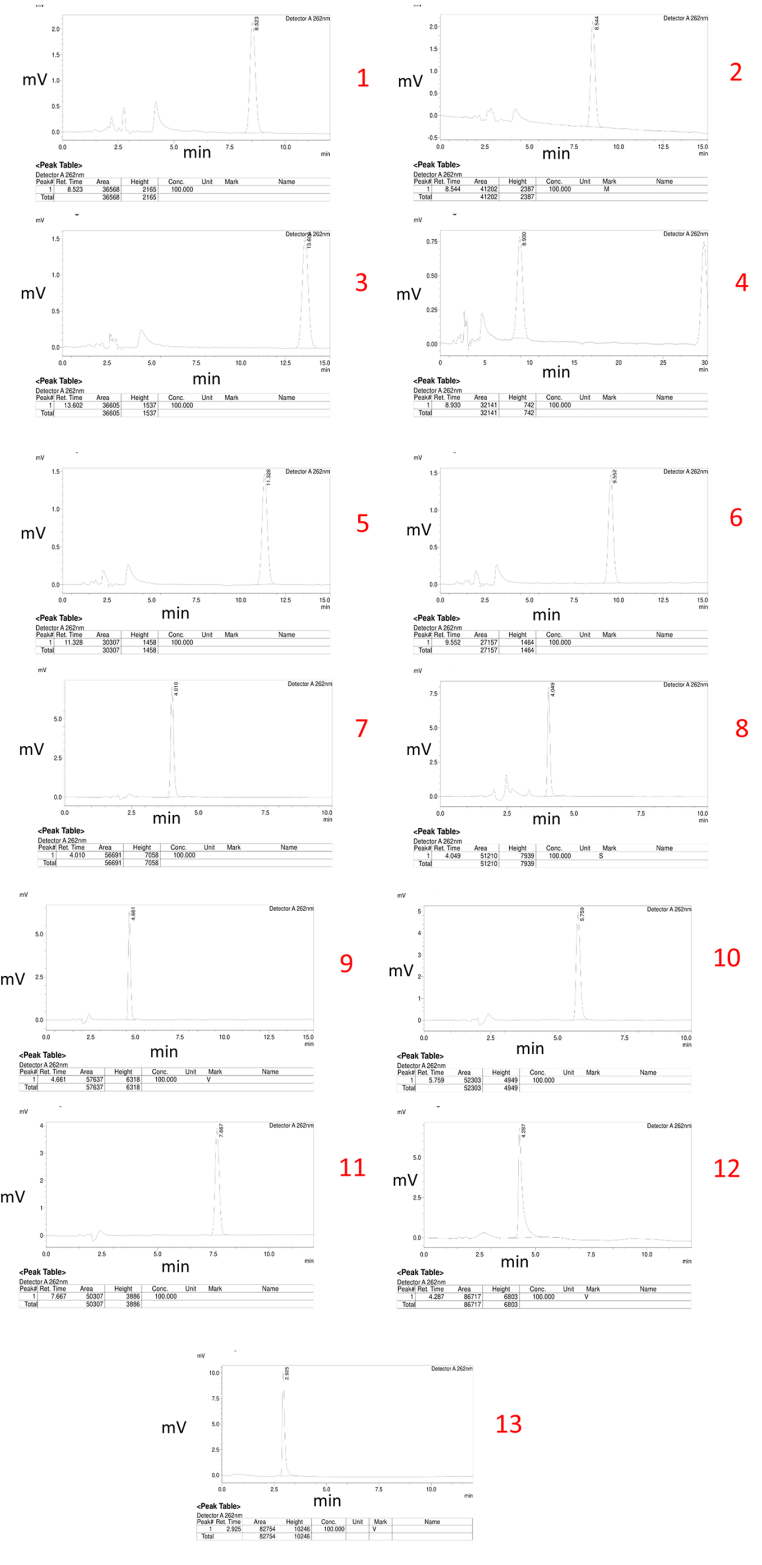
Chromatograms of initial trials of method development.

Trial No. 11 was selected as the optimized method due to its superior performance based on the obtained chromatograms from 13 different trials. The chosen conditions included a specific ratio of the organic phase and mobile phase, as well as a specific flow rate. The optimized method demonstrated excellent peak shape, a high peak area, and a suitable 5-to-8-minutes retention time. This ideal retention time range was considered to be optimal for bioanalytical method development as it allows for the elution of the drug without interference from plasma interferences that typically arise within the first 5 minutes while avoiding excessive consumption of mobile phase beyond 8 minutes, which can increase the cost of the method. Figure 2 shows the different chromatograms of initial trials of method development.

### Optimized Method (Trial 11) Conditions

Stationary Phase: Phenomenex Hyperclone C18 Column

Mobile Phase: Acetonitrile: 10mM Phosphate Buffer pH 6.8

Mobile Phase Ratio: 55:45

Flow Rate: 1 ml/min

Mode: Isocratic elution

Column Temperature: 25 ^o^C

Detection Wavelength: 262 nm

Total run time of the instrument: 10 minutes

### Validation of the optimized HPLC method

**Figure 3.**
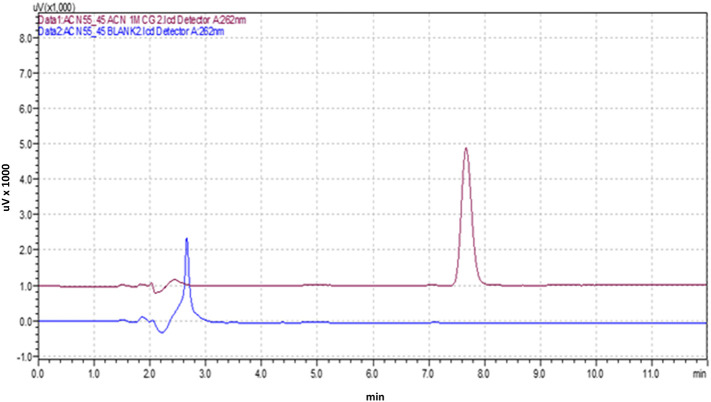
Chromatogram of the Posaconazole with Blank.

The optimized HPLC method was validated according to the ICH Q2 (R1) guidelines, which involved a range of experiments to evaluate the method's performance under different conditions. Figure 3 shows the optimized chromatogram of the Posaconazole.

### Specificity

The developed HPLC method exhibited high specificity in determining Posaconazole, as indicated by the absence of interferences at the retention time of 7.634 minutes for pure Posaconazole and 7.691 minutes for the Posaconazole present in the marketed formulation. This suggests that the method is capable of accurately identifying Posaconazole in the presence of other components.

### Linearity

**Figure 4.**
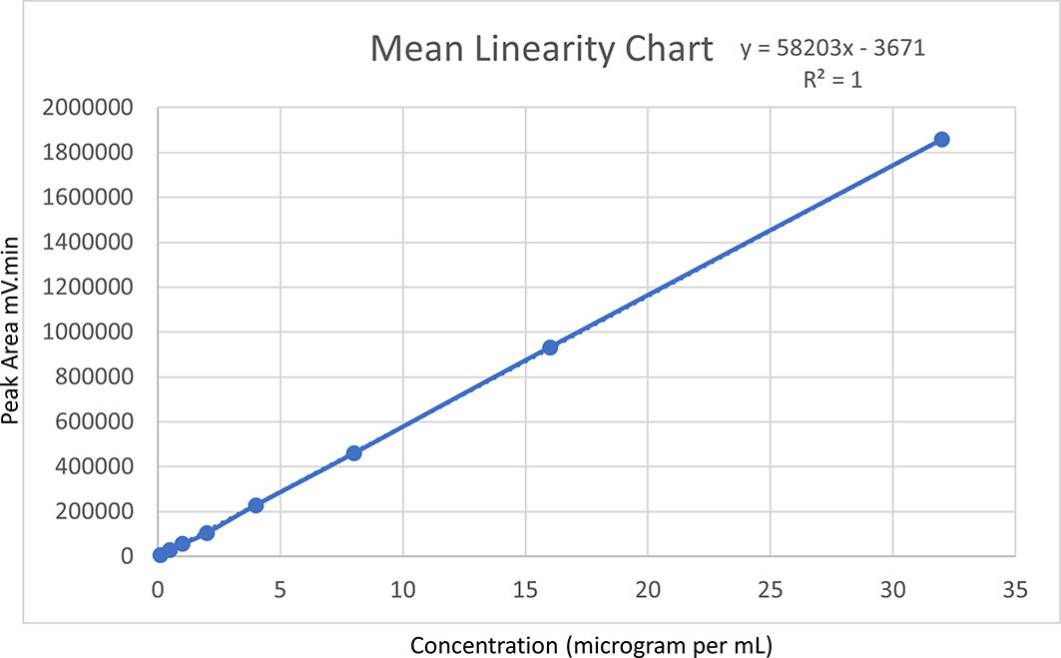
Mean linearity chart.

Posaconazole was quantified in quintuplicate for a concentration range of 0.1 to 32 μg/mL. Individual linear plots were generated for each trial, and the mean value was computed. The plotted linear regression graph showed a linear regression equation of y = 58203x-3671 for the mean value, with a coefficient of determination (R ^2^) of 1. Notably, none of the consecutive R ^2^ values from the five linear regression plots was less than 0.999. This indicated that the optimized method was highly linear within the Posaconazole concentration range, and further experimentation was warranted. Figure 4 represents the linearity of the method via a linear graph.

### Accuracy

Intraday and interday analyses were conducted to assess the accuracy of the optimized HPLC method for quantifying Posaconazole. In the intraday analysis, sextuplicate injections were made at three different Posaconazole concentrations ranging from 2 to 8 μg/mL, while in the inter-day analysis, duplicate injections were made at the same three concentrations. The mean percentage recovery for each concentration was calculated and found to be 91.86%, 89.22%, and 87.46% for 2 μg/mL, 4 μg/mL, and 8 μg/mL, respectively, in the intraday analysis. Similarly, the mean percentage recovery for the inter-day analysis was 94.71%, 89.15%, and 88.09% for the same concentrations. The results showed that the mean recovery percentage for both intraday and inter-day analyses of Posaconazole was within the range of 85%-115%, indicating the optimized method's accuracy and suitability for the precise quantification of Posaconazole. Data represented in the Table 1 evident the accuracy of the method.

**Table 1.** Accuracy of the method.

Time in hours	Concentration in mcg	Retention time	Area	Linear equation	% Recovery
0	2	7.632	102744	Y=58203x-3671	91.42
0	4	7.638	201361		88.07
0	8	7.633	401422		87.00
13	2	7.641	103262		91.86
13	4	7.639	204050		89.22
13	8	7.639	403561		87.46
26	2	7.644	106575		94.71
26	4	7.642	203875		89.15
26	8	7.646	406513		88.09

### Sensitivity

The detection limit is an important parameter in the analytical method validation process as it determines the lowest concentration of analyte that can be detected with acceptable accuracy and precision. In this study, the detection limit of Posaconazole was calculated based on the residual standard deviation of the regression line and its slope. The limit of detection and limit of quantitation were determined to be 0.24 and 0.74 μg/mL, respectively. This suggests that the developed HPLC method is highly sensitive and can detect Posaconazole even at very low concentrations.

### Precision

Four quality control concentrations of Posaconazole were analyzed in duplicate during intraday and interday experiments to assess the precision of the developed analytical method. The mean peak area of the four concentrations (1.5 μg/mL, 4.5 μg/mL, 34.26 μg/mL, and 64 μg/mL) was calculated for both experiments. The results showed that the percentage relative standard deviation of the peak area for all concentrations was less than 2%, indicating the high precision of the method. This precision study confirms the suitability of the optimized method for the accurate quantification of Posaconazole. Therefore, the developed technique shows excellent potential for the precise quantification of Posaconazole, offering a reliable method for pharmaceutical research and analysis. Data represented in the Table 2 evident the precision of the method.

**Table 2.** Precision of the method.

Concentration in mcg	0 Hours		13 hours		26 hours		%RSD	
	Retention time	Area	Retention time	Area	Retention time	Area	Retention time	Area
1.5	7.632	77441	7.641	78642	7.644	79113	0.08	1.01
4.5	7.638	194388	7.639	196151	7.642	197540	0.03	0.81
34.26	7.633	1695217	7.639	1718915	7.646	1732927	0.09	1.11
64	7.635	3213814	7.634	3253934	7.643	3233874	0.07	0.62

### Robustness

To assess the robustness of the developed HPLC analytical method, triplicate injections of Posaconazole at a concentration of 2 μg/mL were performed while varying the operating conditions such as the pH of the buffer, the acetonitrile ratio in the mobile phase, the column oven temperature, the wavelength, the flow rate, and the injection volume. The results showed that the mean percentage relative standard deviation (%RSD) of peak area and retention time were less than 2% for all the varied conditions. This indicates that the optimized methodology has a high degree of robustness, ensuring precise and accurate quantification of Posaconazole even in the presence of small variations in the operating conditions. Data represented in the Table 3 evident the robustness of the method.

**Table 3.** Robustness of the method.

Condition	Retention time	Area	Standard retention time	Standard area	%RSD of retention time	%RSD of area
+2 nm Wavelength	7.641	102225	7.636	104890	0.04	1.82
-2 nm wavelength	7.628	102709			0.07	1.49
+0.2 ml Flow Rate	7.443	107548			1.81	1.77
-0.2 ml Flow Rate	7.851	102052			1.96	1.94
+0.2 pH	7.611	105729			0.23	0.56
-0.2 pH	7.698	105444			0.57	0.37
+2 mcl Injection Volume	7.637	107884			0.01	1.99
-2 mcl Injection Volume	7.635	102026			0.01	1.95
+2 ^o^C Column Temperature	7.633	103537			0.02	0.92
-2 ^o^C Column Temperature	7.634	103159			0.02	1.18
+2% Mobile Phase	7.594	103500			0.39	0.94
-2% Mobile Phase	7.688	104414			0.48	0.32

### System suitability

The suitability of the analytical system for the intended application of Posaconazole quantification in its bulk and dosage forms was evaluated by injecting Posaconazole at a concentration of 1 μg/mL in sextuplicate and analyzing the retention time and mean peak area. The consistent results obtained from the sextuplicate trials indicated the system's suitability for precise and accurate quantification of Posaconazole in its various forms. Data represented in the Table 4 evident the system suitability of the method.

**Table 4.** System suitability of the method.

Flow rate	Rt	Area	NTP	HETP	TF 5%	TF 10%	K factor
1	7.634	55012	5910	25.382	1.132	1.107	1.62
1	7.637	55825	5797	25.874	1.133	1.105	1.62
1	7.635	55936	5848	25.65	1.135	1.105	1.62
1	7.635	55970	5858	25.605	1.132	1.103	1.62
1	7.636	56191	5766	26.014	1.135	1.106	1.62
1	7.637	55842	5828	25.736	1.131	1.102	1.62
%RSD	0.015861	0.727357	0.859556	0.856041	0.147689	0.168548	0.03

### Application of the developed analytical method for quantification of Posaconazole in novel nanoformulations and marketed product

The analytical method developed for quantifying Posaconazole was successfully applied to the practical analysis of Posaconazole in a marketed tablet formulation (Picasa GR 100, INTAS, India). This practical application demonstrated the feasibility and accuracy of the developed HPLC method in quantifying Posaconazole in real-world samples, indicating its potential as a reliable and efficient tool for quality control and assessment of Posaconazole-containing pharmaceutical products. The results highlight the effectiveness of the developed method in analyzing Posaconazole in dosage forms, affirming its potential as a valuable analytical tool for pharmaceutical industries.

### Quantification of Posaconazole in the marketed formulation

The validated HPLC method was employed to determine the concentration of Posaconazole in the marketed tablet formulation (Picasa GR 100, INTAS, India). A standard stock solution of 10 μg/mL of Posaconazole was prepared and introduced into the HPLC system for analysis. The retention time and mean peak area were carefully recorded and analyzed to determine the concentration of Posaconazole. The analysis results indicated that the mean peak area and retention time of POSA were consistent with the anticipated values, which is a strong indication of the reliability of the analytical method. Moreover, the recovery percentage obtained from the analysis was 99%, signifying high precision and accuracy. These observations demonstrate the effectiveness of the HPLC method in determining the concentration of Posaconazole in the marketed formulation with high levels of reliability, precision, and accuracy.

## Discussion

In this study, we successfully developed and validated a reverse-phase high-performance liquid chromatography (HPLC) method to accurately quantify Posaconazole in bulk and dosage forms. Accurate measurement of Posaconazole is crucial for ensuring the quality, safety, and effectiveness of pharmaceutical formulations. Our method addresses this need, offering a reliable and efficient approach for quality control and assessment of products containing this widely used antifungal drug.

To ensure the reliability and robustness of our developed HPLC method, we applied specific acceptance criteria for the validation parameters. These acceptance criteria were as follows:

1. Specificity: The absence of interference in the retention time of posaconazole served as the acceptance criterion for specificity. This ensured that the developed method accurately identified and quantified posaconazole without any interference from other components.
2. Linearity: We assessed linearity using the coefficient of determination (R ^2^) value, with an acceptance criterion of R ^2^ greater than 0.999. This demonstrated a strong linear relationship between the analyte concentration and the corresponding peak area, ensuring accurate quantification across a range of concentrations.
3. Accuracy: Our acceptance criterion for accuracy was set at 85%-115%. This range ensured that the measured concentrations of posaconazole using our method fell within an acceptable percentage deviation from the true or expected values, indicating the method’s ability to provide accurate results.
4. Precision: For precision, we set the acceptance criterion as a percentage relative standard deviation (%RSD) of the peak area not exceeding 2%. This criterion evaluated the method’s repeatability and reproducibility by assessing the consistency of results obtained from multiple injections of the same sample.
5. Robustness: In terms of robustness, we used %RSD of area and retention time as acceptance criteria, with a maximum limit of 2%. This criterion evaluated the method’s ability to withstand deliberate variations in experimental conditions, while maintaining consistent and reliable results.

Our method exhibited excellent linearity within a concentration range of 2-20 μg/mL, meeting the acceptance criterion for linearity (R ^2^ > 0.999). The accuracy of our method fell within the predefined acceptance range of 85%-115%, demonstrating its ability to provide accurate quantification. The precision results, with a %RSD of the peak area below 2%, confirmed the method’s repeatability and reproducibility. Additionally, the method showed robustness by maintaining consistent results within the accepted %RSD limits for area and retention time.

One of the key advantages of our HPLC method lies in its simplicity and efficiency. With a straightforward sample preparation procedure and a relatively short analysis time, our method is practical for high-throughput analysis in diverse laboratory settings. Utilizing a commercially available HPLC column and a well-optimized mobile phase composition ensures optimal elution of Posaconazole.

Moreover, our method offers high sensitivity and accuracy. It demonstrates excellent linearity across a wide concentration range, facilitating precise quantification of Posaconazole in different sample matrices. The method’s accuracy is supported by the close agreement between the measured concentrations and known reference standards.

While our method shows robustness and reproducibility, we acknowledge certain limitations. Specifically, we did not investigate the potential impurities or contaminants that could interfere with the analysis. Exploring impurity profiling and assessing potential interferences would provide a more comprehensive understanding and enhance the method’s applicability.

Future studies could focus on employing different extraction and purification techniques. These efforts would further enhance the reliability of our method and broaden its potential applications.

The repeated analyses we conducted yielded highly consistent results, underscoring the reproducibility of our method. With its precision and accuracy confirmed through comparison with known reference standards, our method proves reliable for accurately quantifying Posaconazole. The comprehensive validation and repetition steps we undertook instill confidence in the method’s robustness and reliability across different experimental settings.

While our method demonstrates robustness and reproducibility within the tested conditions, it is important to evaluate its performance when applied to different instruments or laboratories. Method transferability and inter-laboratory comparisons should be considered to ensure broader application and standardization.

By providing a reliable and efficient means of analyzing Posaconazole, our developed method significantly contributes to the quality assurance and assessment of pharmaceutical products containing this drug. This is particularly valuable for ensuring the therapeutic effectiveness and safety of these formulations, ultimately benefiting patient care and treatment outcomes.

In summary, our study successfully developed and validated an HPLC method for accurate Posaconazole quantification. The method’s reliability, simplicity, and high sensitivity make it suitable for widespread use in various pharmaceutical formulations and matrices. By adhering to specific acceptance criteria, we have established a robust and reliable method that meets stringent standards for specificity, linearity, accuracy, precision, and robustness.

## Conclusion

In conclusion, this study successfully developed and validated a reverse-phase HPLC analytical method for quantifying Posaconazole in bulk and dosage forms. The method showed excellent linearity, precision, accuracy, and specificity. The study adhered to the CONSORT guidelines, ensuring the validity and reproducibility of the results. This analytical method can be used for quality control and assessment of Posaconazole-containing pharmaceutical products, thereby contributing to the development of safe and effective drugs for patients.

## Data Availability

FigShare. Linearity Data of Posaconazole HPLC estimation. DOI: https://doi.org/10.6084/m9.figshare.22331650 This project contains the following underlying data: Linearity Data of Posaconazole HPLC estimation
a. The above-mentioned dataset represents the linearity of the develop HPLC method for Posaconazole estimation. Data are available under the terms of the Creative Commons Zero “No rights reserved” data waiver (CC0 1.0 Public domain dedication).
