# Supplementary material for: Advancing posaconazole quantification analysis with a new reverse-phase HPLC method in its bulk and marketed dosage form
Source: F1000Res. 2023 Jun 27;12:468. Originally published 2023 May 4. [Version 2] doi: 10.12688/f1000research.132841.2 (PMC10314186; doi:10.12688/f1000research.132841.2)
Supplement: Peer review history

# Peer review: Reviewer response for version 2

**DOI:** 10.5256/f1000research.152016.r181903

**Published:** 2023-06-30

**Authors:** Vivek Dave

**Affiliations:** 1 Department of Pharmacy, School of Health Science, Central University of South Bihar, Patna, Bihar, India

Now the article has been accepted in this revised current form.

Is the rationale for developing the new method (or application) clearly explained?

Yes

Is the description of the method technically sound?

Yes

Are the conclusions about the method and its performance adequately supported by the findings presented in the article?

Yes

If any results are presented, are all the source data underlying the results available to ensure full reproducibility?

Yes

Are sufficient details provided to allow replication of the method development and its use by others?

Yes

Reviewer Expertise:

Pharmaceutics, NDDS

I confirm that I have read this submission and believe that I have an appropriate level of expertise to confirm that it is of an acceptable scientific standard.

# Peer review: Reviewer response for version 2

**DOI:** 10.5256/f1000research.152016.r181902

**Published:** 2023-06-27

**Authors:** Praful Balavant Deshpande

**Affiliations:** 1 Teva Pharmaceuticals, Waterford, Ireland

Satisfied with the author response and this article can be accepted without further changes.

Is the rationale for developing the new method (or application) clearly explained?

Yes

Is the description of the method technically sound?

Yes

Are the conclusions about the method and its performance adequately supported by the findings presented in the article?

Yes

If any results are presented, are all the source data underlying the results available to ensure full reproducibility?

Yes

Are sufficient details provided to allow replication of the method development and its use by others?

Yes

Reviewer Expertise:

Pharmaceutical Research & Development

I confirm that I have read this submission and believe that I have an appropriate level of expertise to confirm that it is of an acceptable scientific standard.

# Peer review: Reviewer response for version 1

**DOI:** 10.5256/f1000research.145793.r172005

**Published:** 2023-05-24

**Authors:** Vivek Dave

**Affiliations:** 1 Department of Pharmacy, School of Health Science, Central University of South Bihar, Patna, Bihar, India

The manuscript describes the development and validation of a new reverse-phase HPLC method for quantifying posaconazole in bulk and marketed dosage forms. The study was conducted following ICH guidelines, and the results demonstrate the effectiveness of the developed method in accurately quantifying posaconazole over a concentration range of 2-20 μg/mL. Overall, the manuscript is well-written and organized, with clear descriptions of the methods and results. However, there are a few minor issues that should be addressed before indexing:

1. The authors should provide more information on the sources of posaconazole used in the study, particularly the marketed tablet formulation. For example, what brand or manufacturer was used, and was the formulation within the expiration date?
2. It would be helpful if the authors could provide more information on the selectivity of the developed HPLC method. For example, were there any potential impurities or contaminants that could interfere with the analysis, and if so, how were they controlled for?
3. In Table 3, the % RSD values for precision should be expressed to two decimal places.
4. The authors should consider expanding the discussion section to further emphasize the significance of the study's findings.
5. Finally, there are a few minor grammatical errors and typos throughout the manuscript that should be corrected before indexing.

Overall, I believe this manuscript presents valuable insights into the quantification of posaconazole and provides an important contribution to the field of pharmaceutical analysis.

Is the rationale for developing the new method (or application) clearly explained?

Yes

Is the description of the method technically sound?

Yes

Are the conclusions about the method and its performance adequately supported by the findings presented in the article?

Yes

If any results are presented, are all the source data underlying the results available to ensure full reproducibility?

Yes

Are sufficient details provided to allow replication of the method development and its use by others?

Yes

Reviewer Expertise:

Pharmaceutics, NDDS

I confirm that I have read this submission and believe that I have an appropriate level of expertise to confirm that it is of an acceptable scientific standard, however I have significant reservations, as outlined above.

# Response

**Published:** 2023-06-23

**Authors:** Annamalai Rama

**Affiliations:** Pharmaceutics, Manipal Academy of Higher Education, Manipal, Karnataka, India

Dear Reviewer,

 Thank you for taking the time to review our manuscript titled "Development and Validation of a Reverse-Phase HPLC Method for Quantifying Posaconazole in Bulk and Marketed Dosage Forms." We appreciate your valuable feedback and have made the necessary revisions to address your concerns. Here is a detailed response to each of your points:

 1. Sources of Posaconazole: We have now provided additional information regarding the sources of posaconazole used in our study, including the brand or manufacturer of the marketed tablet formulation. We have also mentioned that the formulation used was within the expiration date.

 2. Selectivity of the Developed HPLC Method: We have included a discussion section in the manuscript that provides more information on the selectivity of our developed HPLC method.

 3. Precision Values in Table 3: We have revised Table 3 to express the %RSD values for precision to two decimal places, as per your suggestion.

 4. Expanded Discussion Section: We have expanded the discussion section of the manuscript to further emphasize the significance of our study's findings. We have provided a more comprehensive analysis of the results and their implications, highlighting the potential applications and benefits of the developed method.

 5. Grammar and Typographical Errors: We have carefully reviewed the entire manuscript, addressing any grammatical errors and typos. We utilized Grammarly Premium University Version to ensure the accuracy of the language and to enhance the overall quality of the manuscript.

 We would like to express our gratitude for your insightful comments and assure you that we have taken them seriously. We believe that the revisions we have made have significantly improved the manuscript.

 Thank you once again for your time and expertise in reviewing our manuscript.

# Peer review: Reviewer response for version 1

**DOI:** 10.5256/f1000research.145793.r172003

**Published:** 2023-05-18

**Authors:** Praful Balavant Deshpande

**Affiliations:** 1 Teva Pharmaceuticals, Waterford, Ireland

The manuscript presents a new reverse-phase HPLC method for quantifying posaconazole in bulk and marketed dosage forms. The study was well-designed and executed, and the results demonstrate the effectiveness of the developed method in accurately quantifying posaconazole within a concentration range of 2-20 μg/mL. Overall, the manuscript is well-written and organized, with clear descriptions of the methods and results. However, there are a few minor issues that should be addressed before indexing:

1. The authors should provide more information on the validation parameters used in the study, particularly the acceptance criteria for accuracy and precision.
2. In Table 2, it would be helpful if the authors could provide a brief explanation of the abbreviations used for the mobile phase and detection wavelength.
3. The authors should consider adding a brief discussion of the advantages and limitations of the developed HPLC method compared to other methods for quantifying posaconazole.
4. It would be helpful if the authors could provide more information on the reproducibility of the method.
5. Finally, there are a few minor grammatical errors and typos throughout the manuscript that should be corrected before indexing.

I believe this study offers valuable new perspectives on posaconazole quantification and represents a significant contribution to the field of pharmaceutical analysis. With only minor revisions required, I recommend Approving the manuscript for indexing.

Is the rationale for developing the new method (or application) clearly explained?

Yes

Is the description of the method technically sound?

Yes

Are the conclusions about the method and its performance adequately supported by the findings presented in the article?

Yes

If any results are presented, are all the source data underlying the results available to ensure full reproducibility?

Yes

Are sufficient details provided to allow replication of the method development and its use by others?

Yes

Reviewer Expertise:

Pharmaceutical Research & Development

I confirm that I have read this submission and believe that I have an appropriate level of expertise to confirm that it is of an acceptable scientific standard.

# Response

**Published:** 2023-06-23

**Authors:** Annamalai Rama

**Affiliations:** Pharmaceutics, Manipal Academy of Higher Education, Manipal, Karnataka, India

Dear Reviewer,

 Thank you for your thoughtful review of our manuscript titled "Development and Validation of a Reverse-Phase HPLC Method for Quantifying Posaconazole in Bulk and Marketed Dosage Forms." We greatly appreciate your positive feedback and your valuable suggestions for improvement. We have carefully addressed each of your points, and we are pleased to inform you that we have made the necessary revisions. Here is our detailed response:

 1. Validation Parameters: We have now provided additional information about the validation parameters used in the study, specifically the acceptance criteria for accuracy and precision. We have included this information in the discussion section, which allows readers to better understand the reliability and robustness of our developed HPLC method.

 2. Explanation of Abbreviations in Table 2: We have made the required changes as per your suggestion.

 3. Advantages and Limitations of the Developed HPLC Method: We have included a brief discussion in the manuscript that highlights the advantages and limitations of our developed HPLC method compared to other methods for quantifying posaconazole. This addition will help readers appreciate the unique features and potential applications of our method.

 4. Reproducibility Information: We have addressed your suggestion and provided more detailed information on the reproducibility of our method in the discussion section of the manuscript. We discuss the precision and repeatability of the method and provide insights into the robustness and reliability of our results.

 5. Grammar and Typographical Errors: We have meticulously reviewed the entire manuscript, correcting any grammatical errors and typographical mistakes. We utilized Grammarly Premium University Version to ensure the accuracy of the language and enhance the overall quality of the manuscript.

 We sincerely thank you for your positive evaluation of our study and your recommendation to approve the manuscript for indexing. We are grateful for your time and expertise in reviewing our work, and we believe that the revisions we have made have significantly improved the manuscript.

 Once again, we extend our gratitude for your valuable feedback and your recommendation to approve our manuscript.
